# Bauxite mining at Atewa Forest Reserve, Ghana: a political ecology of a conservation-exploitation conflict

**DOI:** 10.1007/s10708-020-10303-3

**Published:** 2020-09-24

**Authors:** Sebastian Purwins

**Affiliations:** Institute of Geography, University of Augsburg, 86159 Augsburg, Germany

**Keywords:** Bauxite mining, Atewa forest, Political ecology, Conservation-exploitation conflict, Ghana

## Abstract

Atewa Forest Reserve in the Eastern Region of Ghana represents one of only two reserves with upland evergreen forests in Ghana but is also a possible site for bauxite mining. The Government of Ghana deployed an infrastructure in anticipation for a refined bauxite agreement with China. Ghana’s Government seeks to develop an integrated Bauxite-Aluminum Industry; however, several NGOs try to protect the Atewa Forest and propose that the area should be upgraded to a National Park. In this study, this conservation-exploitation conflict is analyzed from a political ecology perspective elaborating on who are the involved key actors, their relations and what strategies are used. Political ecology is about recognizing the power that actors have at the moment of deciding what, how, and where to conserve nature. Based on interviews done during fieldtrips in 2018, 2019 and 2020 complemented by an analysis of political documents, the identified strategies the NGOs are using in this conflict, can be described as demonstration and upscaling. The aim of this paper is to draw attention on the politicization of nature, in particular Atewa forest reserve and its bauxite resources.

## Introduction

Extraction spaces, even without actually mining taking place yet, are spaces where power relations are destabilized, existing livelihoods are challenged, inequalities emerge and the territorial development is contested. Frequently, these spaces and the processes are marked by tension, friction and accelerated change. Pijpers and Eriksen (2019) point out, that just as the extractive sector is expanding, so is the interest among social scientists in the implications of this expansion. The recent growth in associated research indicates that there is considerable ongoing concern to seek a better understanding of extractive practices and their social, economic, political and environmental effects around the world. For political ecology, a research approach that specifically engages with the causes and consequences of uneven power relations over natural resources and the environment, understanding *conflicts* is a prime focus (Le Billion and Duffy 2018). In addition, political ecology explores the politicization of nature through conflicts. The struggle about bauxite mining at Atewa Forest is an example for a political ecology conflict, where different actors on different scales with different interests compete about a politicized resource.

In 2018, China and Ghana entered a so-called resource-for-infrastructure swap that gained public attention: the Sinohydro Deal. This agreement is viewed controversial, as the repayment shall be done with the revenue of refined bauxite. This requires the development of a bauxite industry and, therefore, further extraction of bauxite. The states of Guinea, Ghana and Sierra Leone are home to the most important bauxite mining areas in Africa. In 2014, Guinea, the fourth largest producer in the world, produced 17.3 million tons of bauxite, Sierra Leone 1.16 million tons, and Ghana only about 837,000 tons (USGS 2016). Although Ghana has extensive reserves, the bauxite aluminum industry is not economically significant, compared to Ghana’s main export goods Cacao and Gold. The country exports bauxite in its raw state, imports aluminum oxide, processes it in a smelter and then exports aluminum. This fragmented supply chain, which began in the 1970s, was beneficial to the companies involved, but not in the countries economic interests (Hart 1977). Since 1942, bauxite is mined in only one location (Awaso) in Ghana. Already during the independence of Ghana, there have been plans to develop an integrated bauxite-aluminium-industry, but this was never fully realized. The Atewa Forest in Ghana’s Eastern Region is one of three possible sites where the government of Ghana is seeking to mine bauxite in order to develop an integrated bauxite-aluminium industry. Up to this point, the Atewa forest with its upland evergreen forests has not been exploited. Due to the ubiquitous impending risk of bauxite mining and ecological risks, several NGOs aimed for an institutionalized protection of the forest. However, the current bauxite deal has become a key part of the government plans for a nationwide industrialization and has been framed along with iron and steel as a *Strategic Anchor Industry*.

I observed the conflict from 2017 until 2020 and analyzed the politicization and the involved actors in this setting. In this paper, the central questions are: (1) who are the involved actors in this conflict? (2) What strategies and methods are used by the alliance that is against bauxite mining at the Atewa Forest? To address these focal points, I use a political ecology approach with its actor-orientated perspective. I will outline the political ecology approach in “Political ecology approach and methods”, following a brief overview about the study area and specific methods used (Study area and methods). I then describe how the conflict emerged and how the Atewa Forest with its bauxite was politicized (The politicization of the territory). I will end with elaborating on the strategies of the NGOs fighting mining at the Atewa Forest and examine the counter reactions from the government in the discussion (The conflict, strategies and actor mapping). This contribution highlights the negotiations and frictions between individuals and groups with different agendas, worldviews and aims within the context of mining. While it is too early to say if the movements against mining at Atewa Forest was successful, the central lesson is that the NGOs (using several techniques) are trying to pull the government back into the conflict arena, to avoid that the exploitation may appear as something economical logical and without an alternative to the population.

## Political ecology approach and methods

The conservation of biodiversity is an increasingly challenging endeavor. Hodgson et al. (2019) argue that conservation conflicts currently poses one of the most significant challenges to wildlife and biodiversity across the globe. The geographical overlap between mining sites and biodiversity hotspots often lead to serious social and ecological challenges over the short and long term. In academia, the conflict between conservation and mining is often framed as the *conservation-exploitation dilemma*. There is a vast number of studies about the dilemma between exploitation and conservation (see Butsic et al. 2015; Helwege 2015; Paredes 2016; Gómez-Valenzuelaa et al. 2020). While there are many drivers for conservation conflicts, Beynham-Herd et al. (2018) argue that many are rooted in larger societal issues (such as poverty and inequality), imbalances of power and inappropriate governance processes.

From an extraction perspective, Engels (2016) defines three types of conflicts: (1) conflicts between civil society organizations on the one hand and the state and mining companies on the other; (2) conflicts between trade unions and mining companies; and (3) conflicts between artisanal miners and mining companies. Pijpers and Eriksen (2019) introduce the *mining encounters* approach, understood as the negotiations and frictions between individuals and groups with different agendas, worldviews and aims within the context of mining operations from the early stages of exploration and development to the final phases of closure and aftermath. From the perspective of conservation, conflicts are mostly labeled as a *conflict of interest*. A typical example of a *conflict of interest* could be over a forest resource, where some groups want to harvest trees, and other groups want to preserve the forest as a habitat for specific species (Adams 2015). Similarly, Bonsu et al. (2019) argue conservation-exploitation conflicts are constructed upon a substance, grasped as an issue that comprise the conflict. Hereby, the ‘substance’ is not only understood as a material substance, but also as narratives or different imaginaries of future development. While Engels (2016) emphasis the actor-centered approach, Bonsu et al. (2019) focus on the relationship and behavior of people.

The general analysis about socio-environmental conflicts in protected areas assumes that conservation is a generator of conflicts because its main objective is to separate part of the territory for nature (García-Frapolli et al. 2018). Conservation involves making choices about the relations between people and nature. If a forest is protected, it is not available for farmers, hunters or loggers (Adams 2015). Castro and Nielsen (2003) argue that conflicts arise because the people or institutions have differences or incompatibilities between their interests, values, power, perceptions and objectives about something in particular. Political ecology poses a different framing of the understanding of socio-environmental conflicts. According to Le Billon (2015: 598), *“political ecology is about politics, and about recognizing the political character of environmental and resource issues”.* In addition, political ecology is about recognizing the power that actors have at the moment of deciding what, how, and where to conserve (García-Frapolli et al. 2018). It is important to highlight that political ecology explores the politicization of nature through conflicts, instead of naturalizing the conflicts through environmental analysis. Political ecology rejects the hypothesis that with greater environmental scarcity or lack of resources there is an increase in conflicts. Rather it assumes that all human decisions, and therefore also conservation, are inherently political (Adams 2015). Robbins (2004) highlights that political ecology views social/environmental change and emerging conflicts with a normative understanding. Political ecology studies have developed several conceptions of ecological conflicts. Socio-ecological conflicts can be defined as struggles associated with the unequal access to, distribution of, and control over natural resources as well as ecological benefits and risks (Le Billon 2015; Martinez Alier 2009; Peet and Watts 2004; Pichler 2016; Turner 2004). Poststructuralist political ecologists have criticized this understanding of conflict and power, arguing for a more relational understanding of conflicts and power that evolves in assembled networks (Bennet 2010; Rocheleau 2015). Schmidt et al. (2019) highlights the strength of a political ecology approach, because (i) it integrates political and ecological dimensions as well as material and discursive elements, (ii) it calls for a normative perspective and (iii) must be more understood as lenses through which conflicts can be analyzed rather than as a theory or method.

In this paper, a political ecology approach is used to look at the bauxite mining Atewa Forest conflict. Robbins (2004: 12) defines such approaches as *“an empirical, research based exploration to explain linkages in the conditions and change of social/environmental system, with explicit consideration to relations of power”.* Bryant and Bailey (1997, p 39) define power as the *“ability of an actor to control”* the access to nature and natural resources as well as the access of other actors to these resources. The paper follows the approach formulated by Schmidt (2013): Identifying the key actors and their relations, the different scales on which they act and which interests they follow. The aim is to elaborate on the politicization of nature, in that case the forest reserve and its bauxite reserves.

## Study area and methods

**Fig. 1:**
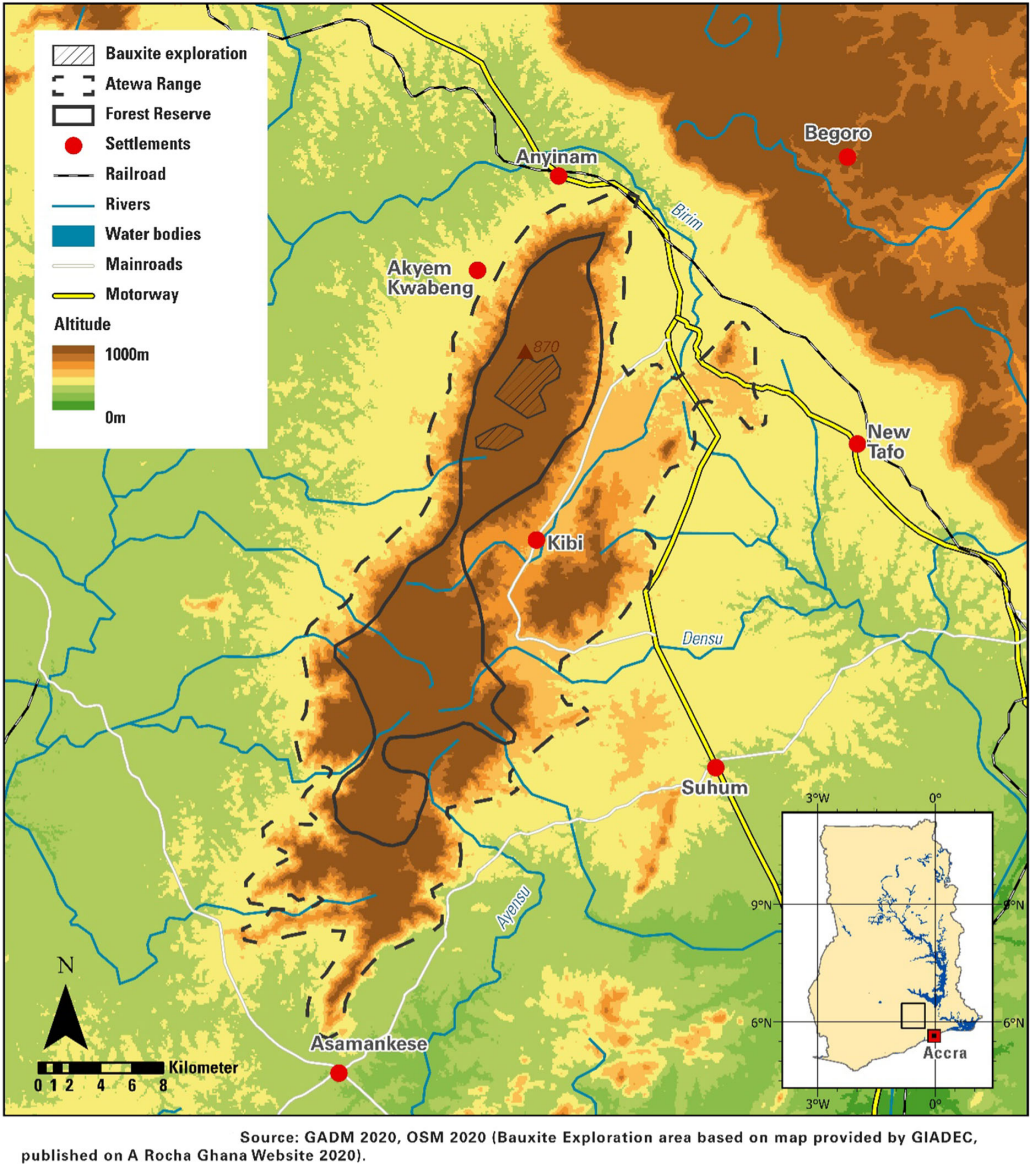
Study Area: Atewa Forest Reserve

The Atewa Range (see Fig. 1) is an ecologically important forest reserve (17,400 hectares) established in 1926. Since that time, Ghana has lost roughly 80% of its forested habitat (Cleaver 1992). Ownership of the reserve is vested in the President of Ghana, while the entire reserve falls within the jurisdiction of the Akyem Abuakwa Traditional Area (McCullough et al. 2007). The head of this area is known as Okyenhene, which is the title of the king of Akyem Abuakwa, an ancient kingdom in the Eastern Region of Ghana (with the capital Kyebi or also written Kibi). The chieftaincy is officially accepted in Ghana. Politicians ask chiefs/kings for advice and permissions because usually they are closer to the people.

Since the Atewa Range is declared a forest reserve, some communal rights are granted: For example, farming within the reserve (admitted farms), collecting forest products (including building materials, canes, vines, ropes, pestles, palm trees, snails, mushrooms, chewing sticks, medicinal plants, game and wildlife), receiving a share in timber royalties resulting from forestry on privately owned land, accessing sacred places, establishing hunting camps and washing for gold (McCullough et al. 2007). The Atewa Range represents some of the highest forest covered hills in Ghana (along with the hills of the Southern Scarp and the Nyinahin Range (Swaine and Hall 1977). The range peaks at 842 m and runs from north to south. It is characterized by a series of plateaus, which are remnants of a Tertiary peneplain (McCullough et al. 2007). The vegetation within the mountain range is very diverse with elements of upland evergreen forest; in addition, the forest is an important watershed from where three important rivers namely the Densu, Ayensu and Birim originate. Atewa Range Forest Reserve is not only recognized as a watershed but also known to constitute the largest and most intact patch of upland evergreen forest in Ghana (Ayivor and Gordon 2012). The reserve is only one of the two reserves in Ghana with upland evergreen forest (Hall and Swaine 1981; Abu-Juam et al. 2003). Because of its uniqueness, the reserve has changed status over the years as a Special Biological Protection Area in 1994, a Hill Sanctuary in 1995 and as one of Ghana’s 30 Globally Significant Biodiversity Areas (GSBAs) in 1999. In 2001, Atewa was listed as an Important Bird Area (IBA) by BirdLife International (Abu-Juam et al. 2003; Rapid Assessment Program 2007).

However, the conservation of this forest reserve also led to some threats and challenges, like illegal hunting and illegal small-scale gold mining. Intensive farming and illegal logging caused further problems with erosion (Ayivor and Gordon 2012). At the edge of the reserve, gold is mined and the forest harbors one of three possible sites for bauxite mining within Ghana. While legal gold mining takes place close to the border of the reserve, bauxite mining would be taking place at the hills and therefore in the forest reserve (Fig. 1).

As later shown, the conflict started in the beginning of 2017; it got more public attention when Ghana and China signed a Memorandum of Understanding in June 2017, the time when I first started with my research. Since then, I have not only undertaken interviews during fieldtrips, but also collected secondary data like policy documents as well as media reports. During the three years I studied the conflict I conducted fieldtrips in March 2018, March 2019 and March 2020, which were instrumental to get further information about the conflict or in regards to interviewing new actors, who were not considered originally. Interviews were conducted with key actors in this conflict including the leading NGO in this conflict (would add here also Name of the NGO; at the head office as well as the local office in Kyebi), VALCO (a smelting company) and a representative from a community close to the forest. In addition, a group meeting of the concerned citizens of Atewa landscape as participating observation, as well as a group interview with this collective were undertaken. Political documents, statements and press releases from the government and NGO were also taken into account. An actor-oriented political ecology perspective, argues that power is exercised by actors. While actors exercise power, they are also met by various forces of resistance and opposition. Mapping Actors can help to understand the power relations between them. Svarstad et al. (2018) argue, that study the agency of individual actors is important in order to explain injustice and a lack of environmental sustainability.

## The politicization of the territory

Since the very first discovery of bauxite in 1914 in the Atewa Range, and initial attempts to establish an integrated bauxite-aluminum industry in 1924, this region has always been one of three possible sites for bauxite mining in Ghana. In 1942, a mine in the Western Region of Ghana started producing bauxite. The first President of Ghana, Kwame Nkrumah, aspired to develop an integrated bauxite-aluminum-industry in order to achieve not only political sovereignty but also economic independence. As a consequence, a smelter was set up in Tema and the Volta Dam was built. However, the proposed integrated industry was never realized. Since then bauxite remained an economically unimportant resource without any further development in that country. This constellation has protected the Atewa forest from mining activities. In 2012, the Forestry Commission informed the NGO A Rocha Ghana^1^ (thereafter named ARG) that the government had given out concessions to prospect bauxite at Atewa to a national company called Exton Cubic. In response to these news, ARG and some smaller civil groups joined forces and opened a dialogue with the government. In 2013, ARG organized a national summit on Atewa Forest with all the important stakeholders including the Forestry Commission and the Water Resource Commission as well as the Minister of Lands and the Minister of Environment. The main outcome of that summit was that it is important to protect the forest, and that no future government should step in and start mining bauxite there. All the participants agreed that upgrading the reserve to a National Park would prohibit any future government of mining bauxite in the Atewa Forest. The Forestry Commission, the Water Commission as well as the Ministry of Lands started the process to declare Atewa Forest Reserve as a National Park.

The campaign to save Atewa Forest is also part of the Green Livelihoods Alliance. This alliance is supported by the Netherlands Ministry of Foreign Affairs. Therefore, the Dutch Ministry together with IUCN Netherlands initiated a survey together with the local NGOs like ARG named the *‘The Economics of the Atewa forest range, Ghana’* (Schep et al. 2016). The study by Schep et al. (2016) compared four different development scenarios for the forest and concluded, that declaring Atewa Forest as a national park with a supporting buffer zone would result in the highest cumulative value for the region. Because a national park has the strictest regulations, the idea of a buffer zone was considered. Such a buffer zone can ensure that part of the traditional activities of local communities develop in a sustainable manner and still provide economic benefits locally. Therefore, the local support should also be granted as the needs of the population around the forest are addressed with this scenario.

The aim in the mentioned survey was to calculate the value of the forest, so that the proposed protection would be more comprehensible for politicians. The foreword of the report was written by the Ghanaian Minister of Land and Natural Resources, Nii Osah Mills at that time, who expressed his intentions: *“Clearly, we simply cannot continue doing business as usual and to this I reiterate the commitment of the Government of Ghana to designate Atewa Range Forest Reserve as a National Park”* (Schep et al. 2016:7). ARG also prepared a needed justification for the Ministry of Lands and Natural Resources. In 2016, the process of upgrading the Atewa Reserve to a national park had progressed so far that the Ministry of Lands and Natural Resources sent out a letter to the cabinet. However, 2016 was also a presidential election year in Ghana between the two major parties, the National Democratic Congress (NDC; at that time in office) and the National Patriotic Party (NPP). One part of the NPP campaign was committed to establish an integrated bauxite-aluminum-industry in order to create jobs and achieve a higher level of industrialization with bauxite mining playing an important role. ARG was in contact with both parties trying to push their idea of a national park to prevent any mining activities in the forest. By the end of 2016, the NPP won the election and Akufo-Addo got into office. Given this new political landscape, it was necessary for the NGOs to start campaigning against bauxite mining at Atewa. On the one side, the NGO sees huge environmental risks for the forest as well as rivers. Bauxite is extracted on a large surface in open-cast mining, resulting in degradation and environmental pollution. An associated scenario-based impact study by the Netherlands Environmental Assessment Agency (Meijer et al. 2018) concluded that over 50% of forestland could be cleared in a worst-case scenario. In addition, toxic by-products (the so-called red mud) are generated during the further processing and leaching of bauxite. The big concern of local environmental organizations, as well as ARG, is that trace quantities of this red mud could get into rivers. The corrosive caustic soda contained in the red mud would be hazardous to the fauna and humans in the vicinity of the rivers. On the other side, the government argues that an integrated bauxite-aluminum-industry would not only generate jobs, but would also finance infrastructure and act as a driver for a nation-wide industrialization.

In 2017, Ghana and China signed a Memorandum of Understanding that may culminate in the development of a $10 billion bauxite for infrastructure barter (part of it also known as the Sinohydro Deal). President Akufo-Addo (2018:9) said in his speech that marked the 61st anniversary of the country’s independence from Britain 3 months after his inauguration: *“Fellow Ghanaians, we have huge infrastructure needs in the areas of roads, bridges, water, electricity, housing, hospitals, schools, *etc*. The problem has always been where to find the money. However, where there is a will, there is a way. My government is going to implement an alternative financing model to leverage our bauxite reserves, in particular, to finance a major infrastructure programme across Ghana. This will probably be the largest infrastructure programme in Ghana’s history, without any addition to Ghana’s debt stock.”*

Shortly after the 2016 Ghanaian general election, the new government ignored the plans to upgrade Atewa Forest to a national park and rejected the inquiries from the NGOs. Therefore, ARG decided to take the conflict into the public space. The initial twitter post of ARG in January 2017 included a call to the president to save Atewa Forest. However, against the background of the 2017 memorandum that the Government of Ghana and the Peoples Republic of China brokered, the attempts to protect Atewa might appear futile. Even though there was no concrete mining taking place or concession given out following the 2017 memorandum, the dissent between the NGOs and the Government about appropriate strategies for territorial development turned specifically the Atewa forest into a contested territory.

## The conflict, strategies and actor mapping

In Ghana, it is usually common to initially carry out a Strategic Environmental Assessment (SEA), in order to identify the possible impacts of bauxite mining and then select possible sites. The NGOs called for the Environmental Protection Agency to require a SEA. However, the new government refuses to follow this procedure. By the end of March 2019, the government presented the *Ghana Integrated Bauxite and Aluminium Development Authority Act.* The Act sets up the legal framework for an authority to develop and establish the integrated bauxite-aluminum industry. The NGOs claim that the establishment of such an authority was unconstitutional, because the Minerals Commission is in charge of such developments. The Government responded by establishing a cooperation, the Ghana Integrated Aluminum Development Cooperation (GIADEC). GIADEC is in charge for organizing bauxite mining in the country and for setting up plans to build refineries and needed infrastructure. The board of the GIADEC consists of representatives of the integrated aluminium industry, members of parliament, a representative of the Ministry of Finance, the chief from Nyinahini, a representative of the Minerals Commission and a representative of the Association of Ghana Industries. GIADEC has a Chief Executive Officer, who was a former Senior Vice-President of the Dell Corporation. Shortly after the setup of the GIADEC, a deal with the Chinese company Sinohydro was signed about infrastructure development in return for refined bauxite. ARG points out that the agreement with Sinohydro is not specific about the location of the bauxite mining. In fact, part of the agreement states that if the country is not able to extract enough bauxite, the country should explore other options. As a consequence, ARG saw a real opportunity to continue with the protest and negotiate with the new government. Several actions were then undertaken to push the agenda that Atewa Forest will be upgraded to a national park. The coalition of NGOs used several tactics that are outlined below to put further pressure on the new government, which can be differentiated into two trategies: demonstrations and up scaling.

### Demonstrations

Demonstration can be understood in two different ways. Either to *demonstrate* something to someone or in the sense of a protest, as a collective gesture of disapproval, like a march. Marking the World Water Day in March 2018 the *Concerned Citizens of the Atewa Landscape*, represented by civil society organizations, NGOs, Youth Groups, Interfaith Groups, Farmer Based Associations and Opinion Leaders and Community Leaders from the Atewa region organized a 6-day walk. Hereby, leading NGO and organizer of this protest march was ARG. The 95 km long walk started at Kyebi (Atewa Region) and ended at the Jubilee House, the presidential palace in Accra. ARG counted about 150 people during the walk. During the march, the demonstrators pointed out that water resources would be destroyed thereby symbolically referring to the UN Sustainable Development Goal 6 (SDG6; *Water* *and Sanitation*), and also planned the march to arrive in Accra on World Water Day. Following the arrival in Accra, the demonstrators handed a petition to the president. The main argument was mostly built on the UN SDGs, specifically SDG6 to *'Ensure availability and sustainable management of water and sanitation for all'*. Shortly after this the Ghana Integrated Bauxite and Aluminium Development Authority Act was implemented by the government. The campaign generated media attention in Ghana. Several local Newspaper, radio broadcasts, and on social media, the issue of Atewa forest and bauxite mining was discussed and reported.

Besides the walk on Water Day, in January 2019 an exhibition about the Atewa Forest was organized by ARG. The Exhibition took place at the British Council in Accra showcasing visual displays (pictures, drawings) and providing information about the Atewa forest and its services to the community. In June 2019, the Atewa Day of Action: March for Atewa, Forest and Water took place in Accra. The route through the capital Accra ended at Parliament House, where a petition was handed to members of Parliament. In 2020 ARG planned picketing in front of the Forestry Commission but had to cancel it due to the Coronavirus pandemic.

While these actions are generally understood as a public protest, one member from ARG describes these protests as tactics, so that “*the governments knows, that we are still watching them*”. In addition, the aim was to inform the people in Accra that their water sources come from the forest (thereby rising public awareness). However, demonstrating also refers to the idea of showing or presenting something, in order to convince certain groups. In the context of the conservation of the Atewa Forest, these important stakeholders include the communities that live in the surroundings of the forest.

Mostly people compared bauxite mining to small-scale gold mining operating in the region and employing many people. ARG, therefore, organized a trip for stakeholders around the Atewa landscape to Awaso in beginning of March 2018. Awaso hosts the only active bauxite mine (since 1942) and is partly owned by the Chinese Bosai Minerals Group (80%) and the state (20%). The idea of this trip was to demonstrate the impacts and environmental risks that open-cast bauxite mining poses. In addition, they visited the village Awaso and demonstrated that the people there do not have a better living situation or more income due to the nearby mine. A former assembly member and opinion leader explained in an interview, that at first, he supported the mining because he hoped jobs would be created. Some of the people who participated in this tour also filmed and took pictures, which are now presented in their communities. Also, the trip has been key in the formation of the Concerned citizens of Atewa Landscape. The group includes local farmers, youth, women and interfaith groups as well as local opinion leaders. The general idea of this trip was to educate the people about the impacts of mining. In addition, ARG aimed to talk to the chiefs around the Atewa forest. The position and role of Chiefs in Ghana was further strengthened in the 1992 Constitution of the Fourth Republic, acting as powerful leaders of a community. The main functions of chiefs include dispute settlement; codification of customary law; organization of rituals, ceremonies and festivals; custody of stool land; organization of communal labour; and promotion of socioeconomic development. Chiefs’ responsibilities thus include both statutory and non-statutory aspects, such as promoting development (Kleist 2011). To educate and convince chiefs about sustainable development, as also done in the aforementioned trip to Awaso, is therefore an important aspect of the campaign to protect the Atewa Forest.

**Fig. 2:**
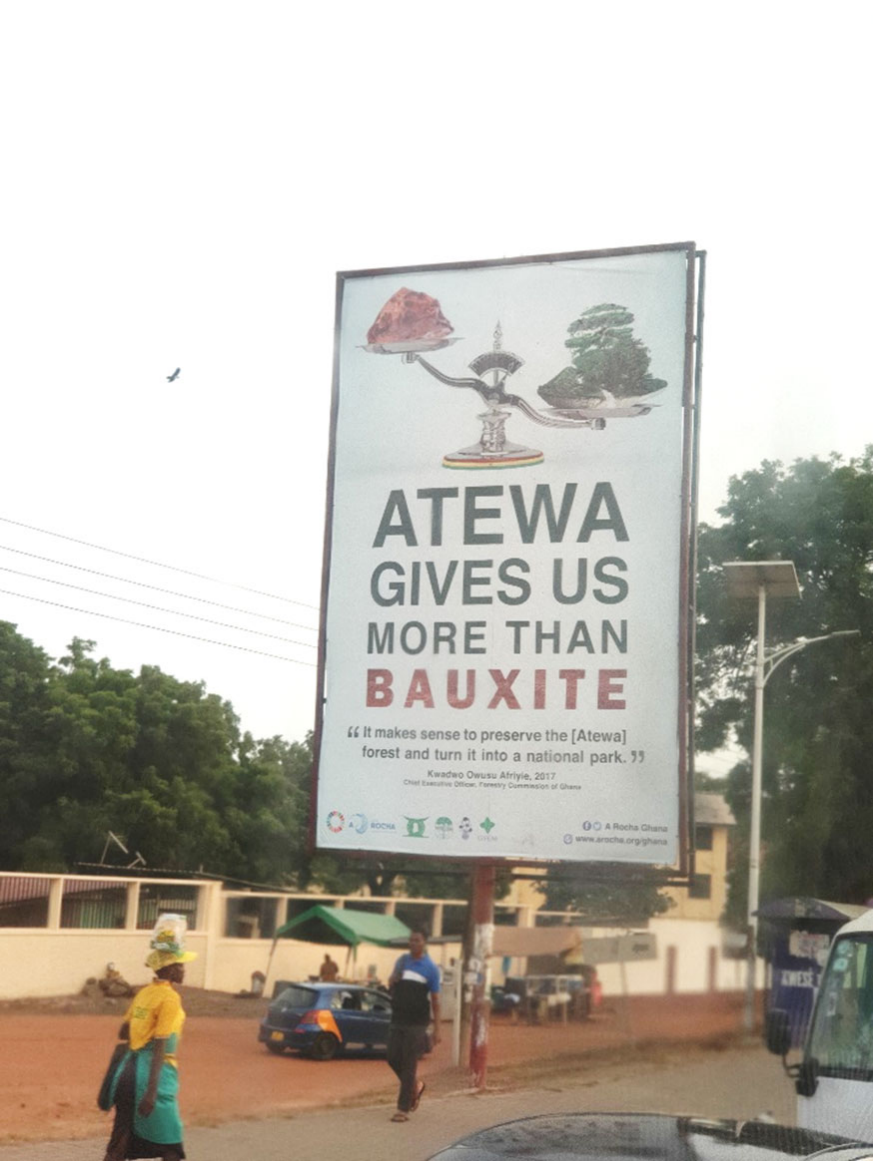
Billboard at the Jubilee House, Accra

In addition, ARG demonstrated their constant watch over the government and exploited a further strategy to rise public awareness in regards to conservation of the Atewa Forest by placing large billboards at strategic locations. While some billboards are located around the forest area in smaller villages, one billboard is placed on the opposite side of the Jubilee House, the presidential palace (see Fig. 2). The billboard is not only placed at a major and highly frequented road in Accra for everyone to see, but also as a statement and symbol of this conflict. It reimagines the mentioned ARG quote that *“the government knows, that we are still watching them”* and is reminding everyday of the unsolved issue.

### Upscaling

During conflicts between social groups, the actors can use a strategy of ‘jumping scale’: upscaling or downscaling (Hogenstijn et al. 2008). Cox (1998) calls this process ‘constructing spaces of engagement’. In this process, actors form temporary coalitions to achieve a common goal. However, these actors can act at different scales. Terlouw (2017) argues that upscaling the conflict arena enables some groups to use their links with powerful groups or individuals to improve their position locally or even on a broader scale. Hogenstijn et al. (2008) are differencing between upscaling the figuration and upscaling the conflict. The latter, is about trying to refer the conflict to a higher spatial scale, where the weak group can achieve a stronger position on the power balance. In contrast, upscaling the figuration means that groups use their links with powerful groups or individuals at higher spatial scales to advance their position locally.

In the context of the preservation of the Atewa Forest, a network on a larger scale is the IUCN Netherlands. Especially because these actors worked together on the prescribed study in 2016. In Mai 2017 the US Ambassador Robert P. Jackson and Netherlands Debuty Ambassador Caecilia Wijge visited the forest. These state officials received a guided tour and were further educated about the importance of protecting the forest by ARG Leading Ghanaian musicians MzVee, Obour, and Sherifa Gunu visit the Netherlands in August 2018 to mobilize with the Ghanaian community to join their plea for the protection of the Atewa Range forest reserve in Ghana. The Ghanaian community in the Netherlands wrote a letter to the president proposing to upgrade Atewa Range Forest into a National Park. International awareness about the Atewa Forest was raised when the famous biologist Edward O. Wilson, an Emeritus at Harvard, Bestselling Author and two-times winner of the Pulitzer Prize for General Nonfiction, wrote a letter to the President of Ghana. The letter was part of a petition led by ARG, but also intended as a critical review of the president. However, more recognized on social media received the tweet from the actor Leonardo Di Caprio in November 2019, who linked an article of the Washington Post about Atewa and called for its protection. In addition, several other international media platforms reported about the conservation efforts of the Atewa Forest including the mentioned article from the Washington Post (2019), Quartz (Asiedu 2018; Oteng-Yeboah 2019) and Foreign Policy (Gbadamosi 2020). While the conflict did receive more international attention in this time frame, the upscale process was limited on rising awareness rather than upscaling the conflict itself. In 2020, the ARG started two attempts to upscale the conflict by a motion during the IUCN World Congress of Conservation in June 2020 and by going to court. Held every 4 years, the IUCN Congress is the world's largest conservation event. The aim is to send a resolution backed by the IUCN to the Ghanaian government to urge them to exclude Atewa as a site for bauxite mining. In January 2020, ARG handed a 30-day moratorium to the government, requesting a statement on the further actions in Atewa Forest. However, ARG received no answer and therefore sued the government for entering the forest in May 2019 and drilling deep holes causing damage to the Forest.

### The two conflict lines and actor mapping

In the beginning of 2020, a youth march around Atewa forest against bauxite mining gained public attention. After the march, several chiefs allied and published a statement against this protest. The two major arguments of this statement are that (1) the forest is already under threats like illegal logging and mining and therefore it is difficult to set up a national park and (2) they dispute, that the NGOs speak for the people around the forest. This opens a new conflict line, between some chiefs of the effected communities trying to delegitimize the protests, the government and the environmental NGOs. Additionally, in June 2019, representatives of the GIADEC visited the Okyenhene, the traditional King of the region. He is also the head of the Akyem Abuakwa traditional council, which at first was sceptic about the mining. However, in their meeting the Okyenhene stressed the need for sustainable mining practices that will ensure the full protection of the environment, but praised the new government for their plans to develop an integrated aluminium industry (Nyabor 2019).

**Fig. 3:**
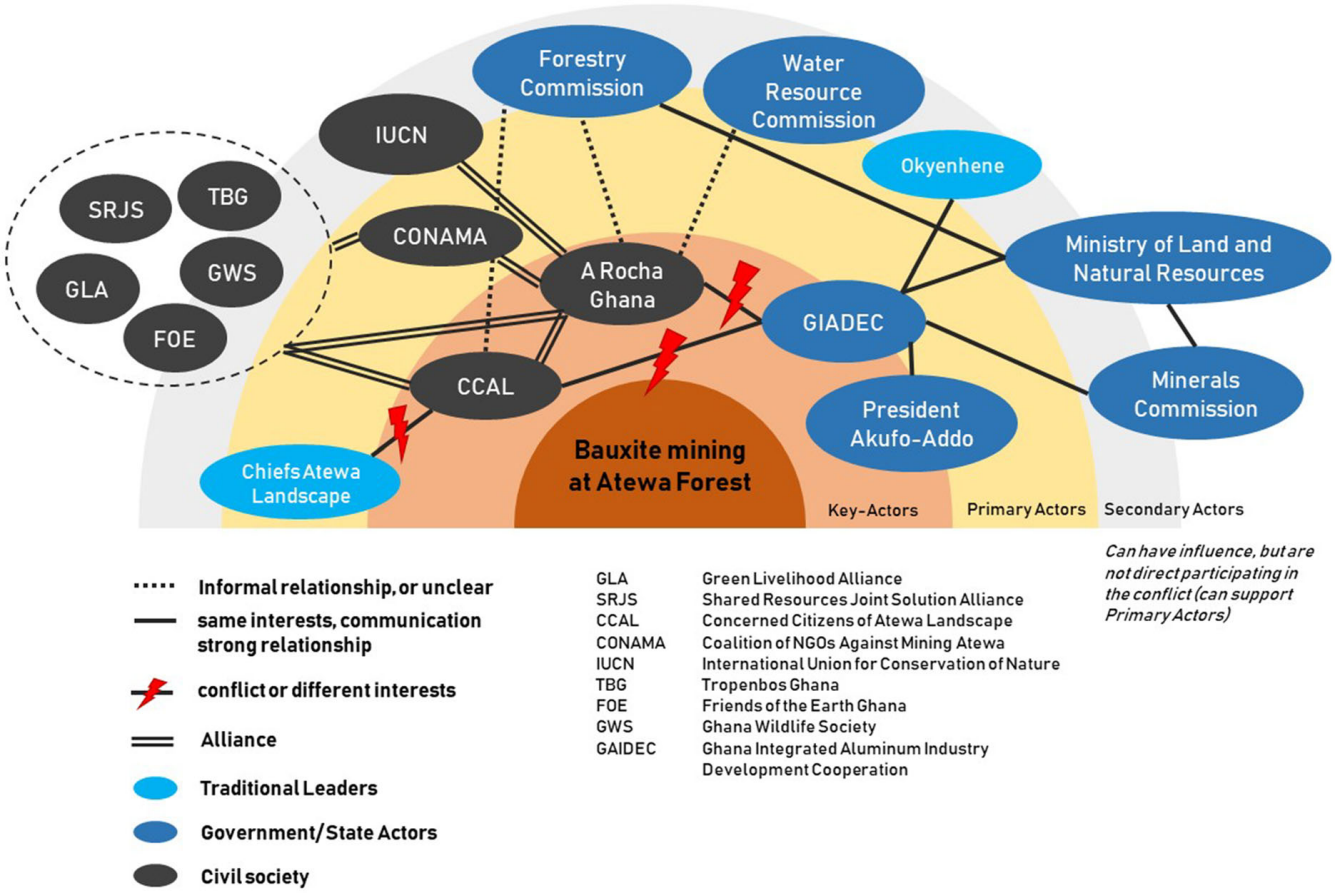
Actor Mapping of the Bauxite Mining at Atewa Conflict

The important actors, divided in key actors, primary actors and secondary actors are put together in a so-called actors map. It sums up the participating actors, their position to each other and enables to identify the conflict lines. However, this mapping only gives an overview over the current status (spring 2020) and leaves out further development. Actors may drop out or appear as well as relations between the actors could change in the future. As argued, Fig. 3 shows that there are two conflict-lines: (a) between the NGOs and GIADC and (b) between the NGOs and chiefs of the Atewa landscape. Because the president is not in a direct exchange or dialog with the NGOs, the conflict is more between GIADC and the NGOs. As explained ARG or other actors address the president directly, but he remains silent and does not speak in this conflict. While he does speak about the future of bauxite mining and the industrialization along with this process, but avoiding discussions about ecological concerns. Having GIADC in charge and negotiating with the NGOs, puts the president more on the sideline of this conflict. The relations between the chiefs and governmental actors remain unclear. However, according to an interview with members of the ARG office in Keybi, the few chiefs are bribed and speaks of elite capturing, which remains an unsettled allegation. It is important to mention, that Keybi, the biggest settlement around the forest, is also the hometown of the President Akufo-Addo. While this was never a particular important argument in the conflict, one member of ARG in Keybi pointed out *“he is one of us, from our town, we should support him. But, at the same time, we are fighting him.”* The relationships between the president and the chiefs are not in the figure. However, it should be pointed out that there might be at least in part some informal relationships between those actors and overlapping interests.

## Conclusion

While some strategies have been more successful than others have, demonstration understood as showing films and taking the local actors to the bauxite mine in Awaso was often described as a successful example. This created greater support for the local population and convinced some political mandate holders from the region about the risks of bauxite mining. As mentioned, an Opinion Leader interviewed changed his mind about bauxite mining after the bus tour to the Awaso mine. Compared to other social movements in the context of mining projects, the NGOs involved in the Atewa Forest conservation formed an Alliance before the current Deal with China was signed. In addition, the movement was in the beginning backed by the government aiming for protecting the forest and turned after the elections 2016 into a movement against the new government. The government of Ghana is silent in the discussion not giving any public statement; however, in one statement by the current president, he declares the concern the NGOs raise, as something technically manageable. Büscher (2010) points out the anti-political tendencies in environmental conflicts, meaning that concerns are reduced to technical and management discussions. Mostly the transformation from a rock into ore is seen as something economical logical to do, because the ore is being translated into benefits for society. However, mining can result in unwanted consequences and these are quite difficult to anticipate or control. Therefore, certain actors produce the idea that extraction is something mostly beneficially. While the new government views the forest as a resource for bauxite, which is symbolized with jobs and industrialization, the environmentalists view the forest as providing essential ecosystem services including unique biodiversity hotspots and clean water. In addition, an illegal hunter would see the forest as source for food and resource to sustain his livelihood. The presented case, framed as a conservation-extraction conflict, is characterized by differences in the idea about nature. Hiding the *political* is also a strategy; however, the NGOs try to pull the new government back into the conflict arena, to avoid that the exploitation may appear as logical and without an alternative to the population. The paper identified main techniques the NGOs uses for this purpose: Demonstration and Up-Scaling. While these strategies where not directly formulated by the NGOs, they all serve the goal of keeping the protest going, gaining more attention and therefore leading to the situation, that the government has to engage with the NGOs because it can no longer ignore or overhear the protest. The paper also elaborated on who the actors are. If the used strategies to protect the forest will be eventually successful is uncertain. The year 2020 marks an election year in Ghana and as ARG pointed out that provides a big chance to put pressure on the current president. At the same time, the outbreak of Covid-19 (SARS-CoV-2) in Ghana in March 2020 is challenging activities like protests and gatherings on both sides of the conflict. Due to these many factors, Atewa Forest will remain a contested territory being constantly renegotiated. Political Ecology is an important approach to deconstruct conflict strategies and drawing attention to the processes of politicization, and how nature is constantly negotiated. In addition, Bridge (2019) calls for a critical engagement with the investment process, from exploration, through development, production and closure. While the conflict between local NGOs and the Ghanaian Government is only one side of the conflict, it remains unclear how direct the involvement of China is in this conflict. Interests between the Governments of China and Ghana may be similar, but in certain aspects could also be contrary. While the idea of bauxite mining in Ghana is nothing new, more an unfulfilled dream, the politicization of Atewa Forest is closely connected to the involvement of China in this conflict. This is because it is the Chinese financial support that may appear as the trigger of this conflict. Further research should therefor also consider not only the conflicts between civil groups and governments, but also what intentions and strategies are competing between a government that hosts natural resources and the Chinese government. Especially against the background of the so-called new scramble for Africa and growing interests of China to secure access to African resources, it will be important to address issues like power relations and how these will have impacts on the local level.

## Footnotes

1 A Rocha Ghana (ARG) is a non-governmental, non-profit environmental organization with a Christian background. A Rocha was founded in Portugal in 1983; the headquarter is now in London and the organization has branches in about 21 countries.
